# Is “Perceived Water Insecurity” Associated with Disaster Risk Perception, Preparedness Attitudes, and Coping Ability in Rural China? (A Health-EDRM Pilot Study)

**DOI:** 10.3390/ijerph16071254

**Published:** 2019-04-08

**Authors:** Janice Ying-en Ho, Emily Ying Yang Chan, Holly Ching Yu Lam, May Pui Shan Yeung, Carol Ka Po Wong, Tony Ka Chun Yung

**Affiliations:** 1 Division of Global Health and Humanitarian Medicine, Jockey Club School of Public Health and Primary Care, The Chinese University of Hong Kong, Hong Kong; janice.ho@link.cuhk.edu.hk (J.Y.H.); may.yeung@cuhk.edu.hk (M.P.S.Y.); 2 Collaborating Centre for Oxford University and CUHK for Disaster and Medical Humanitarian Response, The Chinese University of Hong Kong, Hong Kong; hollylam@cuhk.edu.hk (H.C.Y.L.); wongcarol@cuhk.edu.hk (C.K.P.W.); yungtony@cuhk.edu.hk (T.K.C.Y.)

**Keywords:** water security, disaster risk, risk perception, rural, China, Health-EDRM

## Abstract

Water security is essential for maintaining health and well-being, and for reducing a population’s vulnerability in a disaster. Among resource-poor villagers in China, water-related disasters and climate change may increasingly affect people’s water security. The purpose of this study was to explore the relationship between perceived water security and disaster risk perception in a rural ethnic minority community. A cross-sectional household survey was conducted in 2015 in Xingguang village, Chongqing, China, examining the association between villagers’ perceptions of household water security, disaster risk, and sociodemographic variables. Among 52 household representatives, 84.6% relied on rainwater as their main water source and 63.5% reported having insufficient water on a regular basis. Only 32.7% perceived themselves to be living in a high-risk area, of which climate-related disasters such as storms (44.4%) and droughts (38.9%) were the most frequently reported disasters in their area. Insufficient water quantity, previous disaster experience, and household members on chronic disease medication were found to be associated with higher disaster risk perception. Perceived water security indicators were not found to be predictors of preparedness attitudes and coping ability. Addressing water sufficiency in both disaster risk reduction strategies and long-term water management will be necessary to improve the health and livelihood of rural villagers in the coming decades.

## 1. Introduction

Water is a basic necessity of life and health, with communities requiring the provision of accessible safe drinking water at an affordable cost to meet their basic needs [1]. Lack of access to clean adequate water supplies is an indirect contributing risk factor of disaster through poorer hygiene and water-related diseases that diminish health and increase a population’s vulnerability. On the contrary, adequate water supplies enable households to not only have enough for personal consumption and hygiene but also to build livelihoods, invest in opportune projects, and facilitate community development and poverty eradication [2] (pp. 5, 22). This capacity “to safeguard sustainable access to adequate quantities of acceptable quality water for sustaining livelihoods, human well-being and socio-economic development” is also known as water security [1]. Maintaining water security in communities not only protects health and sustains development but can also help reduce a population’s vulnerability and increase the resilience of households when disaster inevitably strikes. Water-related disasters comprise 90% of the world’s natural disasters [3]. Disasters such as floods, droughts, and storms can greatly affect communities in their development, livelihoods, and health. At the intersection of health and disaster risk reduction is the emerging field of Health Emergency Disaster Risk Management (Health-EDRM), which emphasizes the need to understand the direct and indirect health risks of hazards and to use multisectoral approaches to strengthen evidence-based guidelines and build community health resilience [4]. Health risks in emergencies and disasters should be minimized through a systematic analysis and management of risks across the entire disaster cycle, which should include considerations of the following aspects: “(1) hazard and vulnerability reduction to prevent and mitigate risks, (2) preparedness, (3), response, and (4) recovery measures” [5]. In terms of water, if a community already experiences water insecurity prior to a disaster, this problem will be magnified when sudden or slow-onset disastrous events occur, thus affecting the vulnerability status, preparedness, response, and recovery in a disaster. Inadequate water security and unreliable water systems could compound disaster risk [6,7,8] and have a direct effect on disaster response if water systems fail when they are most needed for survival after a disaster [8] and for emergency healthcare and infrastructural services [9]. A community’s water security is also vulnerable to disaster impacts, as water systems or sources may be damaged by the disasters [6]. Especially with climate change, which will disrupt the hydrological cycle and heighten the frequency and intensity of hydro-meteorological hazards [10], the water security of many communities will be left increasingly at risk unless they take adaptation measures.

Disaster risk perception is an intuitive judgement made by lay persons and may be divergent from objective scientific assessments of disaster risk [11]. In terms of water-related disaster risks such as floods, risk perception research is still in its infancy and many studies take on a non-theoretical approach to risk perception, although measures such as risk characteristics, coping abilities, and preparedness attitudes are often assessed [12]. Research has found risk perception to be affected by prior disaster experience and trust in authorities, with less consistent associations with socio-demographic characteristics, cultural factors, and the influence of media [12,13]. However, environmental conditions and external physical factors have rarely been assessed in relation to a person’s risk perception, and water security has not been suggested amongst such contextual factors.

As securing water at the household level is a cornerstone and foundation to water security [2], there is a need to understand the water insecurity and disaster risk perception at the household and community level. This is particularly true in the context of a rural village, where water systems may be decentralized, thus leaving room for variability amongst different households. Increased reliability and provision of water service can increase the adaptive capacity of a household in the face of disaster impacts and improve household resilience [2]. By understanding the linkage between communities’ water security and disaster risk perception, this can empower the development of water resources management with a consideration towards disaster risk reduction and climate adaptation, contributing to the emergency resilience of the communities [2,14].

As water insecurity problems in a community could be magnified in the occurrence of disasters, this paper seeks to explore the current state of perceived household water security and disaster risk among an ethnic minority village community in Chongqing, China. Perceived water security is measured in this study through self-reported responses adapted from WHO 2011 Guidelines for drinking water quality, 4th Ed. Vol 1 [15]. The aim of this study is to assess the associations of disaster risk perception with perceived water security indicators and socio-demographic characteristics. The study hypothesized that in a rural water-scarce area, perceived water insecurity would be associated with greater disaster risk perception, preparedness attitudes, and coping ability.

Our study was conducted during a health needs assessment trip to Xingguang village in Lutang Township, Pengshui Miao and Tujia Autonomous County, Chongqing Direct-Controlled Municipality, China. In a recent climate change impact assessment, Chongqing was identified as highly vulnerable to future climate change impacts, especially in terms of its water resources [16]. Located in a disaster-prone region, the local community has had frequent exposure to hydro-meteorological risks such as drought, landslides, floods, and storms. The rural village has a largely decentralized water system, and individual households often rely on rooftop collection of rainwater. The village is administratively divided into nine sub-villages and has a total population of approximately 2000 villagers, with average incomes around the international poverty line ($1.90 USD per day per person). It is also ethnically diverse, with minorities such as the Miao (苗族) and Tujia (土家族) ethnicities, as well as the majority ethnicity of Han Chinese.

## 2. Materials and Methods

A pilot cross-sectional stratified sample household survey was conducted in the Xingguang village community in Lutang Township, Chongqing China in February 2015. Households were stratified according to their sub-village and surveys were completed in all nine sub-villages. Within each cluster, we aimed to reach a 10% representation of each sub-village population by simple random sampling. This sampling format is similar to other studies conducted elsewhere [17,18] and enables a representative understanding of different locations throughout the village, limiting the over-accumulation of household surveys from one or two sub-villages. However, this method only takes characteristics of the individual village into account, thus limiting generalizability to other villages. Trained interviewers conducted the face-to-face surveys with each household representative, with the language support of local translators. Each interview took approximately 20–30 minutes. Verbal consent was obtained from all participants. Ethics approval was obtained from the Survey and Behavioural Research Ethics Committee of the Chinese University of Hong Kong. All data were double entered and cleaned by trained staff.

The survey questionnaire of the health needs assessment consisted of 10 sections, with this analysis focusing on the data provided from three sections, namely, (1) respondent background and household characteristics, (2) water and sanitation, and (3) disaster risk perception. The respondent background and household characteristics section included sociodemographic measures such as ethnicity, gender, position as household head, age, education level, occupation, main source of income, annual household income, migrant workers in family, household members with chronic disease, and household members on chronic disease medication. Annual income per capita was calculated by dividing the annual household income by the total number of household members. The water and sanitation section was comprised of general indicators (main source of drinking water, storage of water) and self-reported water security indicators adapted from WHO 2011 Guidelines for drinking-water quality [15]: quality (measured as protection of water source, and boiling of drinking water), quantity (sufficiency of water), accessibility (time needed to fetch water), affordability (money spent on water), and reliability of water (stability of water supply). The outcomes assessed in the disaster risk perception section included the following measures: risk perception (perception of living in a high-risk disaster area), preparedness attitudes (measured as importance of keeping extra medication for a disaster [19], and the necessity of a disaster bag [20]), and coping ability (perceived ability to protect their family’s health and safety in the case of a disaster). The measure of previous personal disaster experience, which was also from this section, was included in the analyses as an exposure.

Descriptive analyses were conducted on the perceptions of water security, disaster risk, and sociodemographic variables. Variables were simplified into binary categorical variables for the univariate analysis. Univariate analyses were conducted using Chi-squared test on the disaster risk perception variables, as associated with subjective water security indicators and sociodemographic variables. A backward stepwise multivariable logistic regression was conducted for each of the disaster risk perception outcomes, which included sociodemographic variables of age, gender, education, and annual per capita income, and variables with *p* < 0.2 in the univariate analyses. After the significant variables were identified in the backward models, the models were re-run while controlling for age and gender. These final models, inclusive of significant variables from the backward logistic regressions and controlled for age and gender, are reported in this paper. Significance level for the multivariable analyses was set at *p* ≤ 0.05. All statistical analyses used SPSS version 20.0 (SPSS Inc., Chicago, IL, USA).

## 3. Results

### 3.1. Descriptive Analyses

Around the time of the survey, Xingguang village was reported to include 540 households and approximately 2300 residents [21]. Our final study sample was comprised of 52 household representatives from the village community. The sociodemographic characteristics of the surveyed households are shown in Table 1. The median household size was five members, and the median annual household income was 20,000 RMB (equivalent to ~$2940 USD). After calculations, the median annual income per capita was 3571 RMB (equivalent to ~$525 USD).

**Figure 1:**
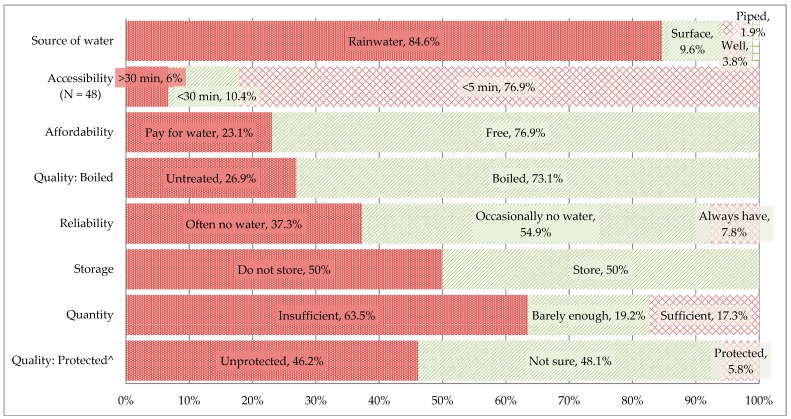
Descriptive statistics of water security indicators adapted from WHO guidelines [15] among households in the Xingguang village community, Chongqing, China (*N* = 52). ^ Protection indicates that the water source quality was protected from runoff water, bird droppings, and animals with a well-lining or casting, and a cover.

Indicators of perceived water security among the surveyed households are shown in Figure 1. Most of the participants used rainwater as their main source of drinking water (84.6%) and reported having an unreliable (37.3% often no water or 54.9% occasionally no water) and insufficient (63.5%) quantity of water. Only 5.8% of respondents had a protected water source and, although half of the households stored their water, less than half of those that stored their water kept the water container covered (38.4%, *N* = 10/26). However, over 70% of the households did not need to use money to buy water (76.9%), had access within 5 minutes (76.9%, *N* = 48), and boiled their water (73.1%).

In terms of disaster risk perception indicators, over half of the respondents (60.8%) reported to have personally experienced a disaster during their lifetime, namely, floods (25.8%), storms (25.8%), snowstorms (22.6%), or droughts (12.9%), among others. However, only 32.7% of respondents believed they were living in a high-risk disaster area, of which storms (44.4%), droughts (38.9%), floods (22.2%), and snowstorms (11.1%) were the most frequently reported disasters in their area. In terms of disaster preparedness attitudes, 47.7% out of 44 respondents agreed to the importance of keeping extra medications for a disaster. Only 34.6% reported the necessity of a disaster bag, although most reported having the following emergency items at home: a flashlight (98.1%), lighter (100%), and emergency blanket (86.5%), with the exception of a whistle (3.8%). Finally, only 34.6% believed they had the ability to protect their family’s health and safety in the case of a disaster.

### 3.2. Multivariable Analyses

Results from the multivariable analyses are shown in Table 2. Perception of living in a high-risk disaster area was found to be associated with insufficient quantity of water (Adjusted Odds Ratio, AOR = 30.48, 95% CI: 1.79–520.47), previous personal disaster experience (AOR = 37.17, 95% CI: 1.44–957.29), and household members on chronic disease medication (AOR = 23.68, 95% CI: 1.49–377.65), after adjusting for gender, age, and annual per capita income. Perceived importance of keeping extra medication for a disaster, as an indicator of disaster preparedness attitudes, was found to be associated with household members with chronic disease (AOR = 4.66, 95% CI: 1.08–20.04), after adjusting for gender, age, and annual per capita income. However, the preparedness attitude of disaster bag necessity was not found to be significantly associated with any predictors. Coping ability, as demonstrated through the perceived ability to protect their family’s health and safety in the case of a disaster, also was not observed to have any significant associations in the backward logistic regression model.

Other perceived water security indicators were not found to be associated with the disaster risk outcomes. Although some perceived water security indicators (such as drinking water source, water storage, and water affordability) were associated with disaster risk outcomes in our univariable analyses, they were no longer associated with disaster risk outcomes in the multivariable analyses.

## 4. Discussion

Overall, our study found that Xingguang village in Chongqing, China, faced vulnerabilities in their perceived water security. The Xingguang village has been obligated to rely on the collection of rainwater as their main source of drinking water despite the low quantity, relatively low reliability, and potential risks of poor quality reported among the majority of the respondents in the village. This indicates a water-scarce context where there is no better alternative source of water resources available. Our findings support the premise that some aspects of perceived water insecurity could further exacerbate the village’s disaster risk perception. Although only one-third of respondents perceived themselves to be in a high-risk disaster area, our findings showed that perceived insufficient water quantity was positively associated with higher disaster risk perception. This demonstrates that within a community that regularly experiences insufficient water quantities, the awareness of their disaster risk is influenced by this external circumstance.

Consistent with previous research, prior disaster experience was found to be associated with a higher perceived disaster risk [12,13,22], while sociodemographic variables, such as age and gender, were not found to be associated [12,13]. Moreover, those on chronic disease medications were more likely to feel at risk of disasters than those who do not take medications, although the preparedness attitude for supplying extra medications was associated with chronic disease persons in general. These results indicate that those who have had relevant experiences related to their vulnerabilities (such as previous disasters or chronic diseases) seem to have greater risk awareness and express more willingness to protect themselves. By enabling more community members to identify their health-related disaster risks and corresponding preparedness actions, this would help lower the vulnerability of the community.

Preparedness attitude (perceived necessity of a disaster bag) and coping ability (perceived ability to protect one’s family in a disaster) were relatively low, with only one-third of respondents in agreement. However, we could not identify any water security or sociodemographic predictors for these two outcomes among a rural population. This indicates that the ability to prepare or cope with disasters is not impaired or impacted by the community’s frequent issues of water insecurity. These two outcomes were also not associated with age or gender, although education might potentially have some effect on coping ability, as it was found to be marginally significant (*p* = 0.088) but did not remain in the final model. Higher risk perception, prior disaster experience, and resources-related variables, such as income, were not found to be associated with preparedness attitudes of a disaster bag, or respondents’ coping ability. As this household survey was conducted during the needs assessment phase of our project, our results indicate the disaster preparedness attitudes and coping ability prior to any disaster education interventions in the community. Thus, increased awareness through a disaster education intervention may be applicable to community members and could be beneficial towards developing an appropriate attitude in regard to disaster preparedness. Additionally, respondents did report possessing common household items that would be useful in the case of a disaster. This indicates that they possess the necessary resources to respond to potential disasters but could be further guided in their awareness and educated on the applicability of their resources.

Our findings on the association between insufficient water and high-risk perception is aligned with the premise that if a community regularly experiences water insecurity then this problem may be objectively exacerbated when sudden or slow-onset disastrous events occur. As such, more should be done to develop the water supply of households in the village, to decrease their vulnerability, and increase their resilience to respond to forthcoming water-related disasters. Literature has shown that those with higher levels of perceived risk are more likely to engage in risk reduction behaviors [23,24]. Households could be guided to better harness the use of rainfall collection, such that they could collect greater quantities of water. Community development should be conducted with the awareness of the risks natural disasters pose to the water supply. Disaster risk reduction activities could be carried out to address this association [25], such as spatial hazard mapping of water insecure households. This may contribute to identifying a vulnerable group in the village that may be more exposed to water insecurity or disaster hazards. Risk assessments can be further conducted to prevent or mitigate the effect of disasters on their water supply. Early warning systems can be put into place for different types of water-related disasters (such as droughts, floods, etc.), such that the villages can ensure a timely response and collect or prepare their water resources sufficiently in the case of emergencies.

Other water security indicators were not found to be associated with disaster risk perception, which supports the argument that adequate water quantity is of greater importance than water quality in water-scare conditions [26]. However, despite the non-significant associations, care should be taken to ensure that the increase of water quantity does not come at the cost of other water security indicators that would further increase the vulnerability of the villagers. Appropriate storage containers could be identified that can maintain the quality of water and protect it from potentially hazardous elements. These actions would in turn not only reduce the disaster risk of the community, but also further enhance the community’s water security.

Our study faced limitations in terms of its cross-sectional study design, small sample size, and missing data. We were unable to include objective assessments of water security indicators, such as water quantity measures or measures of chemical or microbiological contaminants. Additionally, we had no information on respondents’ actual disaster preparedness or coping ability and were only able to assess their attitudes towards it. Our findings cannot be attributed as causation. Excessive amounts of predictor variables would have reduced the power of the analysis, nevertheless we tried to standardize our findings by adjusting for gender and age in the final models. As this was only a pilot study with limited sample size, future research could help tease out possible relationships between water security and disaster risk perception or further identify any associations.

## 5. Conclusions

As far as we know, our study is the first to examine the relationship between water security and disaster risk perception and responses, and our results identified a relationship between insufficient water quantity and disaster risk perception. These findings support the hypothesis that external physical factors, namely inadequate water quantity, can be a contextual factor which affects disaster risk perception. If a community already experiences water insecurity prior to a disaster, its vulnerability will be magnified when affected by sudden or slow-onset disastrous events. With rapid socioeconomic development in China, water security will be challenged and exacerbated. Our study identifies that in a water insecure rural village, perceived insufficient water quantity is associated with higher disaster risk perception, but not preparedness attitudes and coping ability. Water security, particularly sufficiency of water quantity, is an important consideration not only in objective disaster risk assessments but also among disaster risk perceptions and disaster risk management. Particularly in a rural decentralized context, it is essential to consider and develop long-term water management in the lens of disaster risk reduction, as well as disaster risk reduction strategies with an awareness of water sufficiency levels. Such a two-pronged Health-EDRM approach would be valuable to combat future water security challenges and improve the health and livelihoods of disaster-prone rural villages.

## Figures and Tables

**Table 1:** Sociodemographic characteristics of participants and their households in Xingguang village, Chongqing, China (*N* = 52).

Participants	*n*	%	Households	*n*	%
**Gender**			**Main source of household income**		
Male	36	69.2	Farming	25	48.1
Female	16	30.8	Working	25	48.1
**Age**			Others	2	3.8
16–24	6	11.5	**Annual income per capita (*N* = 43)**		
25–44	16	30.8	≤2500 RMB (≤$1 USD/day)	18	41.9
45–64	23	44.2	2501–5000 RMB (≤$2 USD/day)	10	23.3
≥65	7	13.5	5001–7500 RMB (≤$3 USD/day)	7	16.3
**Ethnicity**			>7500 RMB (>$3 USD/day)	8	18.6
Miao	11	21.2			
Tujia	4	7.7	**Household members who are…**		
Han	37	71.2	**Migrant workers**		
**Household Head**			None	5	9.6
Yes	32	61.5	1	25	48.1
No	20	38.5	2	13	25.0
**Education**			3–5	9	17.3
No Schooling	7	13.5	**With chronic disease**		
Primary School	21	40.4	Yes	35	67.3
Middle School	16	30.8	No	17	32.7
High School	5	9.6	**On chronic disease medication (*N* = 47)**		
College or Higher	3	5.8	Yes	22	46.8
**Occupation** ^1^			No	25	53.2
Farmer	21	40.4			
Employee	15	28.8			
Others	16	30.7			

^1^ Other occupations included the following: business person, teacher, student, household work, unemployed, and retired.

**Table 2:** Multivariable analyses of disaster risk outcomes in Xingguang village, Chongqing, China.

Outcome	Variable	Adjusted Odds Ratio (95% CI)	*p*-Value
**Risk perception: Living in a high-risk disaster area (*N* = 46) ^1^**			
	Female	0.51 (0.05, 5.76)	0.584
	45 or older	2.39 (0.24, 24.32)	0.462
	Insufficient quantity water	30.48 (1.79, 520.47)	0.018 *
	Personal disaster experience	37.17 (1.44, 957.29)	0.029 *
	Household member on chronic disease medication	23.68 (1.49, 377.65)	0.025 *
	Per capita income (Ref: >5000 RMB)		
	≤5000 RMB	16.03 (0.92, 278.92)	0.057
	Refuse to answer	1578.1 (6.65, 374,389.16)	0.008 *
**Preparedness attitude: Importance of keeping extra medication for a disaster (*N* = 44) ^2^**			
	Female	0.96 (0.21, 4.34)	0.954
	45 or older	0.48 (0.12, 1.88)	0.289
	Household member with chronic disease	4.66 (1.08, 20.04)	0.039 *
	Per capita income (Ref: >5000 RMB)		
	≤5000 RMB	0.81 (0.17, 3.81)	0.79
	Refuse to answer	0.07 (0.01, 0.98)	0.048 *
**Preparedness attitude: Necessity of disaster bag (*N* = 50) ^3^**			
	Nil		
**Coping ability: Perceived ability to protect in disaster (*N* = 52) ^4^**			
	Nil		

The following variables had *p* < 0.2 in the univariate analyses and were included in the backward stepwise logistic regression models: ^1^ main source of drinking water, water quantity, previous personal disaster experience, and household members on chronic disease medication (or, alternately, household members with chronic disease); ^2^ water storage, ethnicity, occupation, and household members with chronic disease; ^3^ education, previous personal disaster experience, and household members on chronic disease medication; ^4^ water affordability. Each backward logistic regression analysis included age, gender, education, and annual per capita income. * Statistically significant at *p* < 0.05.
